# Can Fyn-Driven Wnt Activation Be the Key to Next-Generation Antifibrotic Therapy in CKD?

**DOI:** 10.34067/KID.0000001087

**Published:** 2026-01-29

**Authors:** Yuanyuan Wang, Dong Zhou

**Affiliations:** Division of Nephrology, Department of Medicine, University of Connecticut School of Medicine, Farmington, Connecticut

**Keywords:** CKD, chronic kidney failure, chronic renal disease, chronic renal failure

Organ fibrosis is the silent architect of chronic disease, ravaging the kidney, liver, lung, and heart and driving a staggering global burden of morbidity and mortality. Despite decades of research, no therapy has yet succeeded in halting or reversing this destructive process, leaving patients to face a relentless march toward organ failure. Currently, US Food and Drug Administration-approved antifibrotic drugs are primarily used for idiopathic pulmonary fibrosis, including nintedanib, a multitarget receptor tyrosine kinase inhibitor (TKI), and pirfenidone, which primarily suppresses TGF*β*/Smad signaling rather than targeting receptor tyrosine kinase (RTKs). Both agents slow the progression of lung scarring, but remain noncurative.

CKD mirrors this therapeutic challenge. Approximately 14% of US adults are affected, yet most remain undiagnosed, and CKD is projected to become the fifth leading cause of death worldwide by 2040. The need for mechanism-driven therapies to halt kidney fibrosis before irreversible damage occurs has never been greater. In this issue, Ma *et al.* identified the Src family non-RTK (nRTK) Fyn as an upstream activator of Wnt/*β*-catenin signaling in fibrotic kidneys. Using saracatinib, a clinically tested TKI, they demonstrated that pharmacologic inhibition of Fyn suppressed downstream *β*-catenin phosphorylation at tyrosine 142, reduced fibroblast activation, and limited extracellular matrix (ECM) accumulation.^1^ While both Fyn and Wnt signaling have been extensively studied,^2^ this phosphorylation event defines a key regulatory node by directly connecting an nRTK to *β*-catenin-mediated nuclear signaling, an interaction not previously well characterized in kidney fibrosis.

The Wnt/*β*-catenin signaling pathway has long stood out as a central regulator in CKD. Its transient activation supports tubular adaptive repair, whereas sustained signaling drives maladaptive fibrosis. However, attempts to globally block Wnt signaling have largely failed because of systemic toxicity and interference with physiological tissue renewal,^3^ reflecting the need for selective modulation. Unlike canonical Wnt regulation through serine/threonine phosphorylation or *β*-catenin stabilization, Tyr142 phosphorylation selectively decouples *β*-catenin from adherens junctions, as observed in pulmonary vascular hyperpermeability under sepsis stress.^4^ This provides a plausible mechanistic explanation for how extracellular stress is transduced into profibrotic programs. Because *β*-catenin undergoes both serine/threonine and tyrosine phosphorylation, each conferring distinct cellular outcomes, targeting specific phosphorylation events thus offer a selective, reversible, and context-dependent approach. Such precision tuning may preserve reparative Wnt activity while suppressing its maladaptive fibrotic signaling. Interestingly, direct evidence of Fyn-mediated *β*-catenin phosphorylation is sparse even in cancer, although the existing studies suggest this interaction may contribute to tumor progression such as colorectal cancer.^5^ From another perspective, this highlights both the novelty of Ma *et al.*'s discovery in kidney fibrosis and the broader potential of targeting Fyn in disease where kinase-driven *β*-catenin activation is pathogenic.

The human kinome, comprising of roughly 518 kinases, orchestrates nearly all cellular functions through phosphorylation. In fibrosis, RTKs, nRTKs, serine/threonine kinases, and mitogen-activated protein kinases act in concert to regulate fibroblast activation, ECM remodeling, and signal integration. RTKs such as platelet-derived growth factor receptor, fibroblast growth factor receptor, and vascular endothelial growth factor receptor stimulate mitogen-activated protein kinase/extracellular signal-regulated kinase and phosphoinositide 3-Kinase/Akt pathways, whereas nRTKs like Src, Fyn, Yes, and Abl phosphorylate *β*-catenin and integrate Wnt/*β*-catenin, TGF*β*/Smad, and Yes-associated protein/transcriptional co-activator with PDZ-binding motif signaling. Src family kinases, including Fyn, therefore represent critical convergence points coordinating multiple profibrotic pathways, making them compelling targets for mechanism-driven antifibrotic therapy. In recent years, TKIs have risen to the forefront as a therapeutic approach capable of halting the molecular progression of kidney fibrosis, bringing renewed optimism to a disease once deemed unstoppable. By targeting kinases such as Fyn, Src, and platelet-derived growth factor receptor, TKIs suppress profibrotic signaling cascades, including Wnt/*β*-catenin, TGF*β*/Smad, and yes-associated protein/transcriptional co-activator with PDZ-binding motif pathways, dampen myofibroblast activation, and reduce ECM accumulation. Agents such as saracatinib, imatinib, and nintedanib illustrate this growing translational promise.

Originally developed for cancer treatment, saracatinib showed limited efficacy in oncology trials. For instance, adding saracatinib to weekly paclitaxel failed to improve outcomes in platinum-resistant ovarian cancer,^6^ and its single-agent activity in estrogen receptor-negative/progesterone receptor-negative metastatic breast cancer was minimal.^7^ Nonetheless, its well-characterized pharmacokinetics and safety profiles make it an attractive candidate for repurposing. Its established clinical record enhances translational feasibility and could expedite entry into early-phase nephrology trials. Preclinical studies have demonstrated that it attenuates hepatic,^8^ pulmonary,^9^ and now renal fibrosis by inhibiting signaling pathways including Fyn,^10^ although the underlying mechanisms differ across organs. In liver fibrosis, saracatinib suppresses hepatic stellate cell activation through the Fyn/focal adhesion kinase/neural Wiskott-Aldrich syndrome protein axis^8^; in pulmonary fibrosis, it broadly reverses fibrogenic pathways, including epithelial–mesenchymal transition, TGF*β*, and Wnt signaling.^9^ In kidney fibrosis, Ma *et al.* demonstrated that saracatinib reduced alpha-smooth muscle actin+ myofibroblast activation, interstitial collagen deposition, and *β*-catenin Tyr142 phosphorylation, thereby preventing *β*-catenin nuclear accumulation without altering total Fyn levels,^1^ suggesting a mechanism based on kinase inhibition rather than protein depletion. Similarly, in primary tubular epithelial cells, Ma *et al.* also showed saracatinib prevented *β*-catenin from translocating into the nucleus,^1^ thus silencing its downstream transcriptional activity. These findings position saracatinib at the intersection of two major fibrotic pathways, tyrosine kinase signaling and Wnt activation, and illustrate how pharmacologic inhibition of Fyn can selectively recalibrate a pathogenic signaling cascade. While these findings demonstrate saracatinib's therapeutic potential, it is important to recognize that dose optimization, patient selection, and context-dependent effects on kinase signaling may influence efficacy in fibrotic diseases.

Despite the findings of Ma *et al.* were promising, several caveats remain. The evidence linking Fyn kinase activity to Wnt/*β*-catenin-driven fibrosis is largely associative rather than strictly causal. Although integrating human CKD biopsies with the unilateral ureteral obstruction model enhances translational relevance, the latter cannot recapitulate functional renal decline, suggesting that the reported biochemical improvements likely reflect attenuation of local injury. Moreover, reliance solely on pharmacologic approach without complementary genetic validation of Fyn, such as knockdown or overexpression, limits mechanistic certainty. Because saracatinib is a broad Src-family inhibitor, off-target effects on other renal or systemic cell types cannot be excluded, including podocytes, where inhibition could potentially affect cytoskeletal dynamics and slit diaphragm integrity; immune cells, where Src family kinases regulate activation and signaling; and tubular epithelial cells where kinase inhibition may influence transport or metabolic function. These caveats highlight the need for careful evaluation of cell type-specific effects and dosing strategies to maximize therapeutic benefit while minimizing unintended consequences. Accordingly, readers should therefore interpret the findings as an important proof of concept that supports targeting Fyn in CKD, but further rigorous mechanistic and long-term studies are needed before clinical translation.

Beyond the specific study of Ma *et al.*, broader challenges persist. CKD is multifactorial and slowly progressive; thus, the long-term effects of Fyn inhibition remains unknown. Moreover, Wnt signaling itself is context-dependent, transient activation promotes repair after acute injury, whereas chronic activation drives fibrosis. The therapeutic goal, therefore, is not complete blockade but precision modulation. In this light, Fyn inhibition represents a refined strategy: intercepting Wnt signaling after ligand engagement but before nuclear transcriptional activation, creating a postreceptor, pretranscriptional checkpoint that allows selective inhibition of maladaptive activity, while preserving reparative functions.

Ultimately, the work by Ma *et al.* exemplified a broader evolution in fibrosis research, shifting focus from static end-stage pathology to dynamic, signaling-driven interventions. Fyn integrates extracellular stress, cytoskeletal organization, focal adhesion turnover, and metabolic cues with nuclear transcriptional programs, linking energy metabolism to fibrotic signaling. Targeting Fyn may therefore recalibrate multiple maladaptive pathways simultaneously. Looking ahead, it is intriguing to speculate that combining Fyn inhibition with complementary agents, such as sodium-glucose co-transporter 2 inhibitors, mineralocorticoid receptor antagonists, or TGF*β* blockers, could amplify renal protection and modify disease trajectory, offering fertile ground for future investigation.

In summary, Ma *et al.* revealed Fyn-mediated *β*-catenin phosphorylation as a critical step in renal fibrosis and demonstrated that saracatinib can pharmacologically uncouple this pathogenic process. This work reframes Wnt/*β*-catenin from a challenging developmental pathway into a druggable circuit in CKD, marking a shift toward next-generation, mechanism-driven precision antifibrotic therapy. The translation of tyrosine kinase biology from oncology to nephrology may soon provide patients with CKD with targeted interventions capable of halting, or even reversing, the fibrotic cascade.
